# Hospital Saturday and Sunday

**Published:** 1882-01

**Authors:** 


HOSPITAL SATURDAY AND SUNDAY.—One of the most worthy charities of New York, is the annual hospital collection, which was taken up on Saturday and Sunday, December 24th and 25th. This charity should appeal to all, for the money thus obtained is used for the benefit of New York's sick and needy, without regard to creed or nativity. Nearly seven thousand of such were treated last year, at the twenty hospitals which share in this collection, and the number is rapidly increasing. This practice only began in 1879, when, not quite twenty-six thousand dollars were collected, but it has met with such general favor, that the amount was increased last year to more than forty-four thousand dollars, and a proportionate increase is expected for the present year. Collections were taken up in all the churches and synagogues, and, in addition, special collection boxes were placed in all the principal retail drug stores, ferry houses, railroad stations, etc. Gifts for special hospitals can be so designated, but a more just distribution is probably obtained by not doing so, for then everything goes into the general fund which is distributed among the hospitals, in proportion to the number of cases treated. It would be well for all large cities to follow this practice.

